# Soil data for mapping paludification in black spruce forests of eastern Canada

**DOI:** 10.1016/j.dib.2018.11.131

**Published:** 2018-11-29

**Authors:** Nicolas Mansuy, Osvaldo Valeria, Ahmed Laamrani, Nicole Fenton, Luc Guindon, Yves Bergeron, André Beaudoin, Sonia Légaré, Mohammed Henneb

**Affiliations:** a Natural Resources Canada, Canadian Forest Service, Northern Forestry Centre, 5320 122 St., Edmonton, Alberta, Canada T6H 3S5; b Institut de recherche sur les forêts, Université du Québec en Abitibi-Témiscamingue, 445 boul. de l’Université, Rouyn-Noranda, Québec, Canada J9X 5E4; c Natural Resources Canada, Canadian Forest Service, Laurentian Forestry Centre, 1055 du P.E.P.S., P.O. Box 10380, Stn. Sainte-Foy, Québec, Québec, Canada G1V 4C7; d Ministère des Forêts, de la Faune et des Parcs, Direction de la gestion des forêts du Nord-du-Québec 645, 1re rue Est, La Sarre, Québec, Canada J9Z 3P3

## Abstract

Soil data and soil mapping are indispensable tools in sustainable forest management. In northern boreal ecosystems, paludification is defined as the accumulation of partially decomposed organic matter over saturated mineral soils, a process that reduces tree regeneration and forest growth. Given this negative effect on forest productivity, spatial prediction of paludification in black spruce stands is important in forest management. This paper provides a description of the soil database to predict organic layer thickness (OLT) as a proxy of paludification in northeastern Canada. The database contains 13,944 OLT measurements (in cm) and their respective GPS coordinates. We collected OLT measurements from georeferenced ground plots and transects from several previous projects. Despite the variety of sources, the sampling design for each dataset was similar, consisting of manual measurements of OLT with a hand probe. OLT measurements were variable across the study area, with a mean ± standard deviation of 21 ± 24 cm (ranging from a minimum of 0 cm to a maximum of 150 cm), and the distribution tended toward positive skewing, with a large number of low OLT values and fewer high OLT values. The dataset has been used to perform OLT mapping at 30-m resolution and predict the risk of paludification in northeastern Canada (Mansuy et al., 2018) [1]. The spatially explicit and continuous database is also available to support national and international efforts in digital soil mapping.

**Specifications table**

**Table:** 

Subject area	*Forestry*
More specific subject area	*Soil Science*
Type of data	*Table, figure and excel table*
How data was acquired	*Organic layer thickness (OLT) data were collected from field measurements across the landscape by manual probing*
Data format	*Raw*
Experimental factors	*No pretreatment of samples*
Experimental features	*Table containing 13,944 measurements of organic layer thickness (OLT) in cm and their GPS coordinates*
Data source location	*Northeastern Quebec, Canada, GPS coordinates for each OLT measurement are available in the excel table*
Data accessibility	*Data are accessible and attached with this paper*
Related research article	Mansuy N, Valeria O, Laamrani A, Fenton N, Guindon N, Bergeron Y, Beaudoin A, Légaré S (2018). Digital mapping of paludification in soils under black spruce forests of eastern Canada. Geoderma Regional. https://doi.org/10.1016/j.geodrs.2018.e00194

**Value of the data**

• In the boreal forests of eastern Canada, organic layer thickness (OLT) is used as a proxy of the paludification process [1].
• Paludification is defined as the accumulation of partially decomposed organic matter over saturated mineral soils, a process that reduces tree regeneration and forest growth.
• Spatially explicit information about OLT is of particular importance in forest management, given the negative effect on forest productivity when OLT is greater than 40 cm.
• Soil organic matter is fundamental to soil and ecosystem functions across a wide range of scales. Continuous data of OLT has numerous applications in sustainable land management, including carbon accounting, soil fertility, biodiversity and forest bioeconomy [2].
• Soil mapping is experiencing a surge of activity with the launch of various international soil programs and networks that are producing digital global soil attribute maps at various resolutions, However, soil data are still currently fragmented and at risk of getting lost if they are not curated [3]. Spatially explicit and continuous OLT database could therefore support national and international efforts in digital soil mapping [4], [5].

## 1 Data

**Fig. 1:**
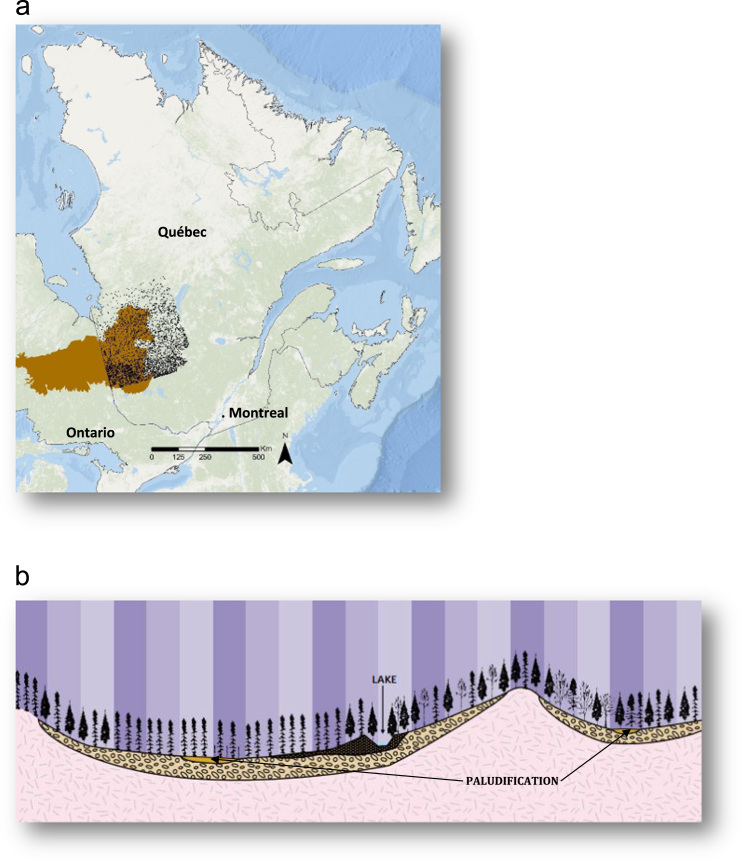
(a) Location of the study area and the sampling sites in eastern Quebec, Canada. The brown area represents the Clay Belt across the province of Quebec and Ontario. (b) Theoretical ecological transect showing paludification in northeastern Quebec (MFFP).

**Fig. 2:**
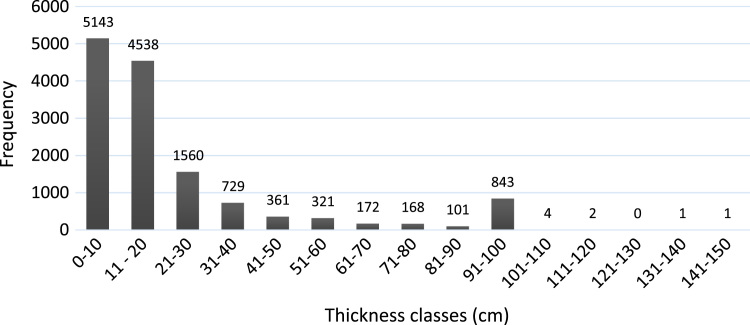
Distribution of organic layer thickness (OLT) values in the dataset (*N* = 13944).

The database contains 13,944 OLT measurements and their respective GPS coordinates that have never been published before. We combined in one unique database multiple OLT measurements from georeferenced ground plots and transects from several previous projects realized in northeastern Quebec (Table 1). The study area covers about 180,000 km^2^ in the province of Quebec in eastern Canada (Fig. 1) and encompasses the black spruce–feather moss bioclimatic domain, which is part of the Boreal Shield ecozone in the south and the Hudson plain ecozone in the north. The landscape of the region is shaped by the draining of the former Glacial Lake Ojibway around 8200 BP, forming the physiographic unit known today as the Clay Belt, which stretches across the Quebec–Ontario border and covers an area of ~ 145 000 km^2^ (Fig. 1). OLT measurements were variable across the study area, with a mean ± standard deviation of 21 ± 24 cm (ranging from a minimum of 0 cm to a maximum of 150 cm), and the distribution tended toward positive skewing, with a large number of low OLT values and fewer high OLT values (Fig. 2). The attached excel table contains the OLT values (in cm) with their respective GPS coordinates (latitude and longitude).

**Table 1:** Sources and descriptions of the organic layer thickness (OLT) dataset from various projects and inventories.

**Projects and inventories**	**Sample Size**	**Sampling area**	**Sampling period**	**OLT (cm) mean ± SD (min–max)**^a^	**Source(s)**
Permanent forest plots	923	Spread across the study area	1972–2007	24 ± 28 (0–100)	MFFP^b^
Temporary plots from 3^rd^ decanal provincial inventory	10,934	Spread across the study area	1992–2002	20 ± 24 (1–100)	MFFP^b^
Ecological observation plots	1 483	Spread across the study area	2008–2014	22 ± 24 (1–100)	MFFP^b^
Villebois paludification study	27	Villebois	2008	35 ± 20 (8–75)	CFS^c^
Villebois paludification study	303	Villebois	2007	30 ± 18 (9–99)	[6]
Valrennes paludification study	132	Valrennes	2010–2014	37 ± 24 (7–150)	[7], [8], [9]
Old forest study	142	Southwest of the study area	2008–2009	41 ± 34 (5–100)	[10]
All datasets	13,944	Spread across the study area	1972–2014		

a SD, standard deviation; min, minimum; max, maximum.

b MFFP, Ministère des Forêts, de la Faune et des Parcs du Québec.

c CFS, Canadian Forest Service.

## 2 Experimental design, materials, and methods

**Fig. 3:**
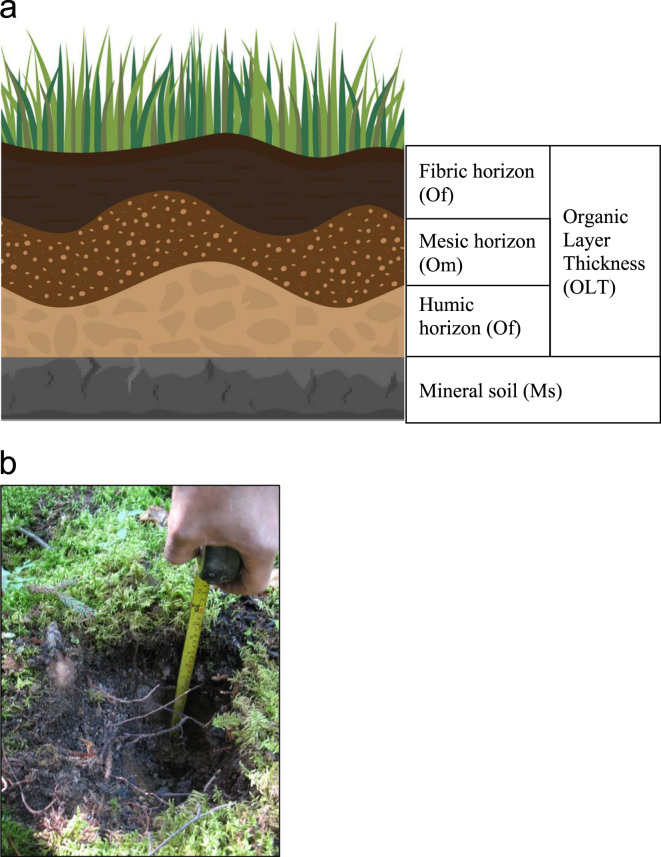
(a) Organic layer thickness (OLT) within the organic horizons and mineral soil, (b) Example of OLT measurement (source: Mohammed Henneb).

Despite the variety of sources, the sampling design for each dataset was similar, consisting of manual measurements of OLT with a hand probe. The method is summarized here but has previously been described in detail [7]. At each sampling point, the hand probe bored through the organic layers until the mineral soil was encountered. For each soil pit, the total OLT was measured as the combination of the fibric horizon, the mesic horizon and the humic horizon (Fig. 3). The whole dataset has been used to perform OLT mapping at 30-m resolution and predict the risk of paludification in northeastern Canada [1]. Before that, subsamples of the database have been used to distinguish and map reversible and permanent paludified landscapes [8] and to measure the effects of mechanical site preparation to mitigate the paludification process in northeastern Canada [9].
