# Supplementary material for: Soil data for mapping paludification in black spruce forests of eastern Canada
Source: Data Brief. 2018 Nov 29;21:2616–21. doi: 10.1016/j.dib.2018.11.131 (PMC6288461; doi:10.1016/j.dib.2018.11.131)
Supplement: Supplementary file 1 — Transparency document. [file mmc1.docx]

Nicolas Mansuy, Ph.D.

Natural Resources Canada

Canadian Forest Service

Northern Forestry Centre

5320 122 st. , Edmonton AB T6H 3S5

Phone : 780-435-7273

Email : nicolas.mansuy@canada.ca

Subject : Conflict of Interest

I , Nicolas Mansuy, declare no conflict of interest with regard to the manuscript intitled: **Soil data for mapping paludification in black spruce forests of eastern Canada**, submitted to the journal Data In Brief .

NM
